# Influence of physical training on intracellular and extracellular zinc concentrations

**DOI:** 10.1080/15502783.2022.2054665

**Published:** 2022-04-03

**Authors:** Víctor Toro-Román, Jesús Siquier-Coll, Ignacio Bartolomé, Francisco J. Grijota, Diego Muñoz, Marcos Maynar-Mariño

**Affiliations:** a Faculty of Sport Sciences Faculty, University of Extremadura, Avenida de la Universidad s/n, Cáceres, Spain; b SER Research Group. Center of Higher Education Alberta Giménez, affiliated to Comillas Pontificial University, Palma Mallorca, Spain; c Faculty of Life and Natural Sciences, University of Nebrija, Campus La Berzosa, Hoyo de Manzanares, Spain

**Keywords:** Platelets, erythrocyte, trace minerals elements, zinc, training

## Abstract

**Background:**

Physical exercise affects zinc (Zn) homeostasis. This study aimed to analyze the influence of physical training on extracellular (serum, plasma, and urine) and intracellular (erythrocytes and platelets) concentrations of Zn.

**Methods:**

Forty young men, divided into a training group (TG; n = 20; 18.15 ± 0.27 years; 68.59 ± 4.18 kg; 1.76 ± 0.04 m) and a control group (CG; n = 20; 19.25 ± 0.39 years; 73.45 ± 9.04 kg; 1.79 ± 0.06 m), participated in this study. The TG was formed by semiprofessional soccer players from a youth category with a regular training plan of 10 h/week. The CG was formed by healthy men who did not practice physical exercise and had not followed any specific training plan. Plasma, serum, urine, erythrocyte, and platelet samples of Zn were obtained and analyzed by inductively coupled plasma mass spectrometry.

**Results:**

The TG showed elevated plasma Zn concentrations (p < 0.01) despite similar intakes. However, TG showed reduced absolute (p < 0.01) and relative (p < 0.05) Zn concentrations in erythrocytes.

**Conclusions:**

Athletes who underwent regular physical training showed elevated plasma and reduced erythrocyte Zn concentrations despite similar intakes to the CG.

## 1. Background

Zinc (Zn) is the second most abundant trace mineral element (TME) in the human body after iron (Fe) [1]. The Zn concentrations are predominantly in skeletal muscle (60%) and bone (30%). Zn plays an important role in different functions of the organism [2] as a cofactor for more than 300 metalloenzymes (enzyme proteins containing metal ions), which can be divided according to their role (catalytic, structural, regulatory, and non-catalytic). These include alkaline phosphatase, lactate dehydrogenase, superoxide dismutase (SOD), carbonic anhydrase, carboxypeptides, and deoxyribonucleic acid (DNA) and ribonucleic acid (RNA) polymerases, among others [3]. Zn regulates intracellular signaling pathways and downstream effects on immune function and redox homeostasis with potential implications for performance and related to metabolic benefits of exercise [4]. Furthermore, Zn promotes proper carbohydrate, protein, and lipid metabolism [5], and is important for optimal cell development, growth, and division, as well as for fertility and hormone metabolism [3,6,7].

TME concentrations are usually under strict homeostatic control [8]. It has been proposed that exercise may induce alterations in Zn homeostasis [9], given that the musculoskeletal system contributes the highest percentage of total body Zn reserves. Zn deficiency has detrimental consequences, especially in tissues with high demand such as skeletal muscle. Specifically, in skeletal muscle it has been observed that Zn affects myogenesis and muscle regeneration due to its effects on the activation, proliferation, and differentiation of muscle cells [10]. Moreover, Zn deficiency has been associated with lower maximal oxygen consumption (VO_2_ max) [11].

Previous studies have evaluated the acute effect of physical exercise on extracellular [9,12] and intracellular Zn concentrations [13,14]. For example, the meta-analysis by Chu et al. [9] observed increases in Zn concentrations immediately after aerobic exercise. On the other hand, Ohno et al. [14] showed decreases in erythrocyte Zn levels after 30 minutes of ergometric exercise. The chronic effect of physical training on Zn concentrations in similar compartments has also been analyzed [4,15–17]. In relation to the above, Maynar et al. [15] observed decreases in serum and urinary Zn concentrations after 6 months of training in elite endurance athletes. Studies evaluating the influence of physical training on Zn concentrations have investigated one [15,16,18], two [13,15,19] or three compartments [20–22]. However, no research have been found analyzing five compartments simultaneously to assess Zn concentrations, which could provide more complete information. In addition, most of the research on the influence of physical exercise on TME concentrations involves cyclic sports [15,23–25], whereas there have been few studies on intermittent or acyclic sports [26,27].

Serum is the most frequently assessed compartment in extracellular Zn status research [4,9,28]. However, it does not seem to be a specific indicator of Zn status in athletes or the general population [29–31]. Factors such as stress, infection, and inflammation could affect serum Zn concentrations [30,32]. Therefore, it is necessary to consider other compartments to assess Zn status. The study of intracellular Zn concentrations is not common in the scientific literature [28]. Previous authors proposed the intracellular determination of TME in erythrocytes because it is not affected by the acute inflammatory response or by short-term diet [33]. In contrast, we think that the study of erythrocyte TME could inform us of past dietary intake (2 to 3 months earlier) because the half-life of erythrocytes is approximately 120 days [34]. As an alternative to assessing intracellular concentrations, we suggest that the study of platelets could provide more current information on dietary intake of the mineral (approximately 2 weeks earlier) because platelets have a half-life of approximately 10 days [35].

Long-term physical training produces different adaptations that could influence Zn redistribution among different compartments and organs [28,36]. This redistribution appears to depend on the type of physical exercise, training status, exercise duration, and environmental temperature [2]. In this regard, we believe that an assessment of Zn status requires more compartments to understand its kinetics and nutritional status as indicated by previous authors [31]. Therefore, the study aimed to analyze the influence of long-term physical training on extracellular (serum, plasma, and urine) and intracellular (erythrocytes and platelets) Zn concentrations and to observe if the concentrations change according to training status.

## 2. Material and methods

The methodology and participants were similar to those described by Toro-Román [37].

### Participants

Forty young men participated in this study. All the participants were informed about the purpose of the study and signed a consent form before enrolling. The protocol was reviewed and approved by the Biomedical Ethics Committee of the University of Extremadura (Cáceres, Spain) following the guidelines of the Helsinki declaration of ethics, updated at the World Medical Assembly in Fortaleza (2013), for research involving human subjects (code: 13/2021). A code was assigned to each participant for the collection and treatment of the samples in order to maintain their anonymity.

The participants were divided into a training group (TG) and a control group (CG). All the participants had been living in the area of Cáceres (Spain) for at least 24 months before the beginning of the research. The TG consisted of 20 semiprofessional soccer players from a youth category belonging to group V of the National Honor Division with a regular training plan of 10 h/week (aerobic-anaerobic training and strength training of 30 minutes twice a week). All of them had been participating in high-level competitions and had trained for at least five years before the experimental period. The CG consisted of 20 young physically inactive healthy men did not practice physical exercise regularly and had not followed any specific training plan in the previous 6 months. The characteristics of both groups are shown in Table 1.

**Table 1.** Characteristics of the two study groups

	CG (n = 20)	TG (n = 20)
Age (years)	19.25 ± 0.39	18.15 ± 0.27
Weight (kg)	73.45 ± 9.04	68.59 ± 4.18*
Height (m)	1.79 ± 0.06	1.76 ± 0.04
Muscle (%)	44.22 ± 5.71	49.03 ± 2.56*
Fat (%)	15.64 ± 5.78	9.32 ± 2.76*
VO_2_ max (mL/Kg/min)	45.61 ± 4.95	61.02 ± 4.35**
VE (L/min)	88.34 ± 11.18	120.56 ± 18.79**
Resting heart rate (bpm)	67.31 ± 6.49	54.41 ± 5.29*
Maximum heart rate (bpm)	189.3 ± 7.1	193.8 ± 6.5
Physical activity (MET-hours/weekly)	27.36 ± 4.45	56.13 ± 6.21**

The participants had to meet the following criteria for their inclusion in the study: must be a male, not have any injuries or illness during the investigation or at least 6 months before the study, and not follow any special diet or take vitamin/mineral supplements, or specific supplementation, medication, or over-the-counter medication. The measurements, sample collection, and Zn determination were carried out at the beginning of the season and were similar to those described by Maynar et al. [15,17].

### Anthropometric measurements

The morphological characteristics of the participants were evaluated in the morning after an overnight fasting period. All measurements were made by the same operator who was skilled in kinanthropometric techniques, under the International Society for the Advancement of Kinanthropometry recommendations [38]. Body height was measured to the nearest 0.1 cm using a wall-mounted stadiometer (Seca 220. Hamburg. Germany). Bodyweight was measured to the nearest 0.01 kg using calibrated electronic digital scales (Seca 769. Hamburg. Germany) in nude, barefoot conditions. A Holtain© 610ND (Holtain, Crymych, UK) skinfold compass, accurate to ±0.2 mm; a Holtain© 604 (Holtain, Crymych, UK) bone diameter compass, accurate to ±1 mm; and a Seca© 201 (Seca, Hamburg, Germany) brand tape measure, accurate to ±1 mm, were used for the anthropometric assessments. The equations of the Spanish Group of Kinanthropometry [39] were used to calculate the muscle and fat percentage. The anthropometric measurements obtained were height, weight, skinfolds (abdominal, suprailiac, subscapular, tricipital, thigh, and leg), bone diameters (bistyloid, humeral biepicondyle, and femoral biepicondyle) and muscle perimeters (relaxed arm and leg).

### Nutritional evaluation

All participants completed a dietary questionnaire to ascertain nutritional intake in the days prior to the assessments. The survey consisted of a 4-day daily nutritional record, of three preassigned week days, and one weekend day. On each day, all participants recorded the amount (in grams) of each food consumed in every meal ingested on every one of the 4-days. Once completed, every questionnaire compiled the total amount of each food consumed, grouped by meals. Then the nutritional composition of their diets was evaluated using different food composition tables [40].

### Physical activity evaluation

Physical activity was assessed using the International Physical Activity Questionnaire – Short Form (IPAQ-SF) Spanish version [41–43]. The questionnaire consists of questions about the frequency and duration of vigorous, moderate, and walking activity. The time spent on vigorous, moderate, and walking activity was weighted by the energy spent to produce MET-hours/week. A trained researcher assisted the participants to respond. The questionnaire was filled out on the day of the assessments.

### Physical performance test

An exercise test was used to evaluate the performance variables after sample collections. The trial consisted of an incremental test until exhaustion on a treadmill (Ergofit Trac Alpin 4000, Germany), equipped with a gas analyzer (Geratherm Respiratory GMBH, Ergostik, Ref 40.400, Corp Bad Kissingen) and a Polar pulsometer (Polar® H10, Kempele, Finland). All tests were performed in the morning (between 11:00-11:30 a.m.) under similar atmospheric conditions (21–24°C and 45–55% relative humidity) after a free breakfast.

All participants ran progressively for 15 minutes to guarantee a warm-up phase before the test, ending at the initial speed of the test. The CG performed 5 minutes at 6 km/h, 5 minutes at 7 km/h and 5 minutes at 8 km/h. The TG ran at 8, 9, and 10 km/h respectively. Then, the participants performed the exercise test. The protocol consisted of running in incremental stages, until voluntary exhaustion, starting at an initial speed of 8 km/h for the CG and 10 km/h for the TG and increasing it by 1 km/h every two minutes, with a stable slope of 1%. In the TG, training intensity and volume were reduced on the two previous days by applying a regenerative load in order to avoid fatigue.

### Sample collection

At eight o’clock in the morning, 12 mL of venous blood was drawn from each subject in fasting conditions, using a plastic syringe fitted with a stainless-steel needle. Plasma, serum, urine, erythrocyte, and platelet samples of Zn were obtained at the beginning of the season. In the TG, training intensity and volume were reduced on the two previous days applying a regenerative load. The preparation of the samples was similar to that described by Maynar et al. [15,17].

For serum, a blood sample of 5 ml was collected in a metal-free polypropylene tube (previously washed with diluted nitric acid) and then centrifuged at 3000 rpm for 15 minutes at room temperature. Serum was aliquoted into an Eppendorf tube (previously washed with diluted nitric acid) and was left to stand at −80°C until further analysis.

For plasma, a blood sample of 5 ml was collected in a metal-free polypropylene tube (previously washed with diluted nitric acid) with ethylenediaminetetraacetic acid (EDTA) and then centrifuged at 1800 rpm for 8 minutes at room temperature. The platelet-rich plasma (PRP) obtained was collected in a metal-free polypropylene tube (previously washed with diluted nitric acid) and centrifuged for 15 minutes at 3000 rpm and the plasma was aliquoted into an Eppendorf tube (previously washed with diluted nitric acid) and was left to stand at −80°C until further analysis. One milliliter of pure water was added to the tube of the concentrate of platelets and stored at −80°C.

The erythrocytes were extracted from the rest of the blood and were washed with 0.9% sodium chloride (NaCl) three times. They were then aliquoted into Eppendorf tubes (previously washed with diluted nitric acid) and conserved at −80°C until biochemical analysis.

The first morning urine sample obtained from all subjects, under fasting conditions, was collected in polyethylene tubes previously washed with diluted nitric acid and frozen at −80°C until analysis.

The remaining 2 ml of blood was used for the determination of red blood cells and platelets using an automatic cell counter (Coulter Electronics LTD, Model CPA; Northwell Drive, Luton, UK).

### Erythrocyte, platelet, plasma, serum, and urinary zinc determination

The technique used for the determination of Zn was similar to that employed by Maynar et al. [15–17].

### Sample preparation and reference material preparation

Prior to analysis, all samples were thawed and homogenized by shaking. Zn analyses were performed by inductively coupled plasma mass spectrometry (ICP-MS). The detection limit was in the range of 1 nmol/L. To prepare the analysis, the organic matrix was decomposed by heating it for 10 h at 90°C after the addition of 0.8 mL HNO_3_ and 0.4 mL H_2_O_2_ to 2 mL of serum, plasma, urine, erythrocytes, and platelet samples. The samples were then dried at 200°C on a hot plate. Sample reconstitution was carried out by adding 0.5 mL of nitric acid, 10 µL of Indium (In) (10 mg/L) as the internal standard, plus ultrapure water to complete 10 mL.

Reagent blanks, element standards, and certified reference materials (Seronorm, lot 0511545, Sero AS Billingstand, Norway) were prepared identically, and used for accuracy testing. Before the analysis, the commercial control materials were diluted according to the manufacturer’s recommendations.

### Sample analysis

Digested solutions were assayed with an ICP-MS Nexion model 300D (PerkinElmer, Inc., Shelton, CT, USA) equipped with a triple quadrupole mass detector and a reaction cell/collision device that allows operation in three modes: without reaction gas; by kinetic energy discrimination with helium as the collision gas; and in reaction mode with ammonia as the reaction gas. Both collision and reaction gases such as plasmatic argon had a purity of 99.999% and were supplied by Praxair (Madrid, Spain). Two mass flow controllers regulated gas flows. The frequency of the generator was free-swinging and worked at 40 Mhz. Three replicates were analyzed per sample. The sample quantifications were performed with In as the internal standard. The linearity of the calibration curves for In in plasma, serum, urine, platelets, and erythrocytes fulfilled the accepted criteria with a value of R^2^ higher than 0.985. The values of the standard materials of this element (10 µg/L) used for quality controls agreed with intra and inter-assay variation coefficients of less than 5%.

### Statistical evaluations

Statistical analyses were carried out with IBM SPSS Statistics 22.0 for Windows (SPSS Inc., Chicago, IL, USA). A p < 0.05 was considered statistically significant. The results are expressed as means ± standard deviations. The normality of the distribution of variables was analyzed using the Shapiro–Wilk test. The homogeneity of the variances was analyzed with the Levene test. Student’s t-test was used to compare the concentrations between both groups. Effect size (ES) was calculated using the *d* of Cohen [44]. ES of 0.2, 0.4, and 0.8 were considered small, moderate, and large, respectively [45].

## 3. Results

The results obtained in this study are shown below. Table 1 details the characteristics of the participants. There were significant differences in weight, muscle percentage, fat percentage, VO_2_max, expiratory volume, resting heart rate, and physical activity levels (p < 0.05).

CG = Control group; TG = Training group; VO_2_ max = maximal oxygen consumption; VE = expiratory volume; MET = Metabolic equivalent of task; * p < 0.05; ** p < 0.01.

Table 2 reports the nutritional intake in both groups. No significant differences were observed.

**Table 2.** Nutritional intakes of both groups

	CG (n = 20)	TG (n = 20)
Energy (kcal/day)	2112.34 ± 345.78	2456.16 ± 504.11
Water (L/day)	1.145 ± 0.241	1.421 ± 0.356
Carbohydrates (g/kg/day)	3.11 ± 1.28	3.98 ± 1.78
Proteins (g/kg/day)	1.25 ± 0.37	1.44 ± 0.41
Lipids (g/kg/day)	1.51 ± 0.47	1.64 ± 0.31
Zn (mg/day)	15.18 ± 8.21	13.58 ± 7.94

CG = Control group; TG = Training group; Zn = Zinc.

Table 3 provides the erythrocyte and platelet values in both groups. There were no differences between groups.

**Table 3.** Erythrocytes and platelets values in both groups

	CG (n = 20)	TG (n = 20)	ES
Erythrocytes (cell 10^12^/L)	4.81 ± 0.72	4.76 ± 0.89	0.14
Platelets (cell 10^9^/L)	190.23 ± 67.13	198.35 ± 60.51	0.17

CG = Control group; TG = Training group; ES = Effect size.

Table 4 shows the extracellular (serum, plasma, and urine) Zn concentrations. TG had higher plasma Zn concentrations compared to CG (p < 0.01).

**Table 4.** Plasma, serum, and urinary concentrations of Zn

	CG (n = 20)	TG (n = 20)	ES
Plasma (μg/L)	1335.44 ± 251.74	1550.56 ± 239.16**	0.63
Serum (μg/L)	804.90 ± 137.59	832.72 ± 123.14	0.15
Urine (μg/L)	438.77 ± 229.45	336.45 ± 163.90	0.37

CG = Control group; TG = Training group; ES = Effect size; ** p < 0.01

**Figure 1.**
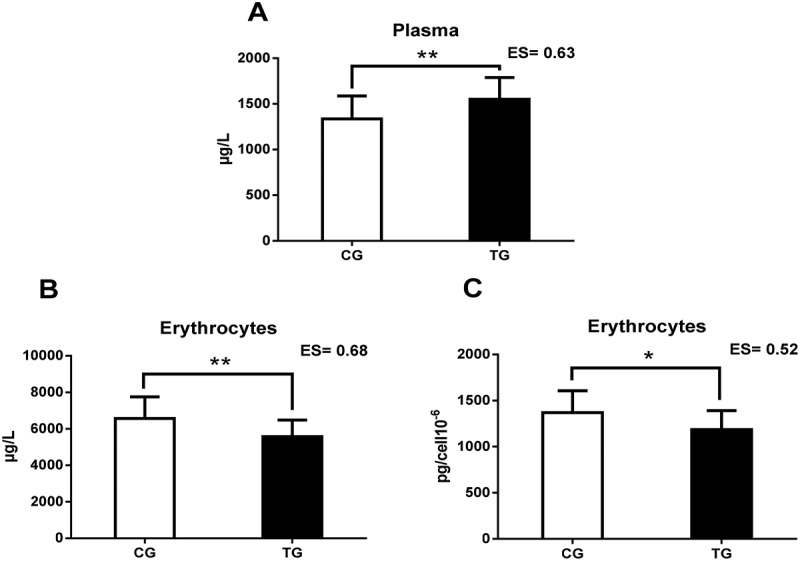
This figure shows the relevant results of the study. A: Plasma Zn concentrations; B: absolute Zn levels in erythrocytes; C: Zn concentrations relative to erythrocyte cells; Zn = Zinc; CG = control group; TG = training group; ES = effect size; *p < 0.05; ** p < 0.01.

Table 5 shows the intracellular (erythrocyte and platelet) Zn concentrations in both groups. TG showed lower erythrocyte Zn concentrations, in both absolute (p < 0.01) and relative (p < 0.05) values (see Figure 1). There were no differences in platelet Zn concentrations.

**Table 5.** Relative and absolute values of Zn in erythrocytes and platelets

	CG (n = 20)	TG (n = 20)	ES
Erythrocytes (μg/L)	6569.33 ± 1185.07	5570.57 ± 908.70**	0.68
Erythrocytes (pg/cell10^−6^)	1368.61 ± 238.45	1185.22 ± 205.39*	0.52
Platelets (μg/L)	377.27 ± 76.72	330.56 ± 98.13	0.38
Platelets (pg/cell 10^−3^)	1.983 ± 0.411	1.666 ± 0.496	0.32

CG = Control group; TG = Training group; ES = Effect size; *p < 0.05; ** p < 0.01

## 4. Discussion

This study showed higher plasma Zn concentrations and lower erythrocyte levels in athletes who performed regular physical training. To our knowledge, this is the first research in athletes to assess Zn concentration in five compartments simultaneously and with the same analytical technique. Based on the results obtained, the assessment of TME should not be limited to extracellular compartments only [8], due to the limitations described for plasma/serum zinc concentration as a marker of Zn status in humans [31]. The relationship between Zn status and serum Zn concentration can be affected by inflammation, hormones, and age [4,30]. Therefore, serum Zn assessment must be complemented with other compartments to describe Zn kinetics. Novel to this study, platelets were obtained to complement the intracellular Zn concentration. As mentioned above, we believe that platelets could provide updated information on intracellular Zn concentrations due to their “brief“ half-life [35]. However, we have not found any studies in athletes that analyze this compartment to assess Zn nutritional status. Table 1 shows the ergospirometric and body composition differences between groups, reflecting the influence of regular physical training.

The Zn concentrations obtained in each compartment were within the ranges reported in other investigations with similar techniques [12,17,46–48]. In addition, the Zn concentrations in the different compartments were determined by ICP-MS, so there is no variability in terms of the analytical instrument used. The differences observed between plasma and serum Zn concentrations could be related to the presence of Zn in coagulation factors [49,50].

Quantification of nutritional intake is important to assess TME concentrations. Lukaski et al. [51] reported that Zn nutritional status, together with other TMEs, could be predictors of physical performance in athletes. Decreased Zn intake is one of the factors that may affect Zn metabolism in athletes [36]. Athletes generally have lower serum Zn concentrations despite higher total dietary Zn intake, requiring higher Zn intake than those who are physically inactive [28]. Table 2 did not report differences between groups in the intakes of macronutrients, water and Zn, so it is assumed that the differences observed could be due to the influence of physical training. Both groups ingested Zn above Dietary Reference Intakes (DRI = 9.5 mg/day) [52]. The involvement of Zn in energy metabolism emphasizes the necessity of an adequate dietary intake of Zn for those who are physically active [2]. As mentioned above, Zn deficiency has been associated with lower VO_2_max, carbon dioxide production, and respiratory exchange rate [11]. Current recommendations for nutritional intake before and after physical exercise are based on high-carbohydrate diets that often include food sources low in total and bioavailable Zn [28]. This would be in agreement with the results obtained in the study where a higher carbohydrate intake and lower Zn intake are observed, without being significant, in the TG. In addition, Zn absorption may be affected by other nutrients, such as phytates, calcium, or tin, which could affect its status [53]. A separate analysis of extracellular and intracellular findings will be presented for a better discussion.

Regarding extracellular concentrations, higher plasma Zn concentrations were observed in the TG in the present study. However, there were no differences in serum and urine Zn concentrations. Rodriguez-Tuya et al. [16] reported that aerobic (cyclist and endurance athletes) and anaerobic (judo and fencing) athletes showed higher Zn concentrations in plasma. However, they did not analyze nutritional intake. Similarly, Koury et al. [54], observed higher Zn concentrations in long-distance athletes compared to triathletes, middle-distance athletes and short-distance swimmers and athletes. However, other studies found no differences between soccer players [55] or swimmers [24] and CG. Savaş et al. [56] observed reduced plasma Zn concentrations after a 6-week aerobic training program in subjects who did not train regularly, in contrast to this study. Similarly, Dressendorf and Sockolov [25] reported lower plasma Zn concentrations in marathon athletes. Likewise, Rakhra et al. [57] reported lower Zn concentrations in people with moderate and high levels of physical activity. In this study, it was observed that training status influenced plasma Zn concentrations.

Previous studies have shown contradictory results in serum. On the one hand, higher concentrations have been observed in anaerobic (judo and speed athletes) and anaerobic-aerobic athletes (football players) [18], as well as in rowers [58], karate athletes, and runners [59] compared to the CG. On the other hand, Chu et al. [28] documented lower serum Zn concentrations in athletes as reported by Maynar et al. [15], Brun et al. [60], in young gymnasts and Arikan et al. [61], in wrestlers. However, Fogelhom et al. [62] found that there were no differences between groups in a sample of over 200 participants (endurance athletes and CG).

Concerning urinary Zn concentrations, Nuviala et al. [59] observed no differences between female athletes of different sports modalities and the CG, as in this study. Similarly, Maynar et al. [15] found no differences in basal levels between athletes and the CG. However, other authors reported higher urinary Zn concentrations after Zn supplementation [63], after strenuous exercise [22,64] or after a repeated heat exposure program [20].

The elevated plasma Zn concentrations observed in the TG could be related to the predominant actions in soccer. Soccer is an anaerobic-aerobic sport where sprints, acceleration, deceleration, changes of direction, and trauma are prevalent [65]. Consequently, these actions lead to increased muscle damage, and increased protein and amino acid catabolism, which could induce a higher release of Zn from muscle cells to plasma, since most Zn concentrations mainly predominate in skeletal muscle [2,21,66]. Elsewhere, Rodriguez-Tuya et al. [16] reported that anaerobic athletes (judo and fencing) had higher plasma Zn concentrations due to the lower role of the antioxidant system and energy metabolism, where Zn acts as an enzyme cofactor, in anaerobic modalities. As an adaptation, aerobic training could increase Zn entry into the cell to promote metalloprotein synthesis, triggering lower plasma Zn concentrations [54].

Regarding intracellular compartments, the TG recorded lower Zn concentrations in erythrocytes, in both absolute and relative values. However, there were no differences in platelets, although they followed a similar tendency. Research on Zn concentrations in erythrocytes reported contradictory results. Fogelhom et al. [62] found higher erythrocyte Zn concentrations in erythrocytes in athletes compared to the CG, who ingested less Zn. Similarly, Singh et al. [19], documented elevated Zn concentrations in female runners. Similarly, Ohno et al. [67], observed higher erythrocyte Zn concentrations in sedentary people after a 10-week aerobic training program. Likewise, De Carvalho et al. [68] reported higher erythrocyte and lower plasma Zn concentrations throughout the season in swimmers, contrary to the results reported in the present study. In relation to the results obtained in the present study, Maynar et al. [17] found that athletes had lower erythrocyte Zn concentrations compared to the CG. Furthermore, Zn concentrations were related to training levels (r = −0.678; p = 0.000). Similarly, Deuster et al. [69] described reduced Zn concentrations in trained female runners. Furthermore, they reported a relationship between Zn intake and Zn concentration in red blood cells. Chu et al. [28] suggested that erythrocyte Zn levels were higher in athletes as a result of possible adaptive effects of regular exercise.

A possible factor for the reduced Zn concentrations observed in the TG could be a reduced Zn intake in previous months. As mentioned above, the half-life of erythrocytes is approximately 120 days [34]. Additionally, the intracellular concentration of TME in erythrocytes is not affected by the acute inflammatory response or by short-term diets [33]. Therefore, the reported data on erythrocyte Zn concentrations could not be recent. However, based on the study of intraplatelet Zn concentration, the Zn status could have been corrected given that the half-life of platelets is approximately 10 days [35], which could provide current information. Another possible factor could be related to the results observed in plasma. Zn acts in erythrocytes as a cofactor for carbonic anhydrase and SOD. Besides, Zn binds to the erythrocyte membrane and hemoglobin [70]. Thus, the hemolysis produced by physical training, specifically soccer [71], could release Zn into extracellular compartments, reducing its concentration [72–74]. Hemolysis increases plasma Zn concentrations as the concentration of Zn in erythrocytes is approximately 10-20 higher than in plasma or serum [75,76]. This would explain the high levels in plasma and the lower concentrations in erythrocytes with respect to the GC.

SOD and carbonic anhydrase are involved in the erythrocyte, playing an important role during physical exercise as an antioxidant. SOD provides a catalytic function for the dissociation of the radical oxygen species, superoxide, into oxygen or hydrogen peroxide [77]. In erythrocytes, oxygen exchange with hemoglobin occurs, causing significant intracellular oxidative stress [78]. Correlations between erythrocyte Zn and SOD activity in athletes increase the requirement of Zn in athletes for antioxidant adaptation [54]. Similarly, carbonic anhydrase is crucial for gas transport and acid-base balance [79]. Previous authors have observed that low Zn intake reduces carbonic anhydrase activity [11]. Therefore, considering the results of this research, we suggest increasing Zn intake in athletes.

We have not found research on intraplatelet Zn concentrations in athletes. It has been mentioned that platelets may disintegrate and release Zn into serum during coagulation [80]. Furthermore, the previous article documented that the increase in serum Zn concentration can be attributed to a slightly increased dilution in plasma, derived from platelets and hemolysis. Recent studies reported that Zn is an important intracellular signaling molecule for platelets as well as a modulator of platelet function [81]. Previous authors observed that Zn regulates cell signaling originating from reactive oxygen species. In addition, reduced intracellular levels of Zn could modify the biochemical properties of signaling molecules [82]. Therefore, adequate Zn intake is required in athletes due to the role of Zn in platelets. Further research is required to confirm these results.

This study has some limitations. First, the small single-sex sample; thus, additional participants are needed to confirm results; second, a cross-sectional study was conducted by obtaining samples at one moment during the sports season in the TG; finally, the lack of complementary Zn data such as Zn transporting proteins, hormonal status, and the indicators of infection or inflammation, and of muscle lysis [29]. The current evidence is inconclusive to determine the relationship between physical training and Zn status in cross-sectional data. The development of longitudinal and cohort studies could clarify this relationship [4].

## 5. Conclusions

Participants who underwent regular physical training showed elevated plasma and reduced erythrocyte Zn concentrations despite similar intakes to the CG.

Muscle damage, caused by physical training, could lead to increased plasma Zn released from muscle cells. The results obtained in erythrocytes could be due to possible nutritional deficits caused in previous months or to hemolysis produced by physical training, which could also increase plasma Zn concentrations.

We suggest performing a multi-compartmental assessment of Zn status due to discrepancies in the assessment of isolated extracellular and intracellular Zn compartments. We think that the assessment of TME in platelets could provide a current evaluation. Therefore, we recommend investigators to analyze this compartment in future studies.

## Data Availability

All data pertaining to the conclusions of the study are found within the article. The corresponding data set used is available under reasonable requests.

## List of abbreviations

CG: control group

EDTA: ethylenediaminetetraacetic acid

Fe: iron

ICP-MS: inductively coupled plasma mass spectrometry

IPAQ: international physical activity questionnaire

MET: metabolic equivalent of task

PRP: platelet-rich plasma

DRI: dietary references intakes

Rpm: revolutions per minutes

SOD: superoxide dismutase

TG: training group

TME: trace mineral elements

VE: expired volume

VO_2_max: maximal oxygen consumption

Zn: zinc
